# Supplementary material for: Oxalic acid blocked the binding of spike protein from SARS-CoV-2 Delta (B.1.617.2) and Omicron (B.1.1.529) variants to human angiotensin-converting enzymes 2
Source: PLoS One. 2023 May 18;18(5):e0285722. doi: 10.1371/journal.pone.0285722 (PMC10194883; doi:10.1371/journal.pone.0285722)

**Appendix**

### S1 Fig. Effects of OA on the interaction between ACE2 and wild type of SARS-CoV-2 Spike RBD. Inhibitory curves of ACE2 binding to wild type of SARS-CoV-2 RBD in the presence of OA determined by ELISA.


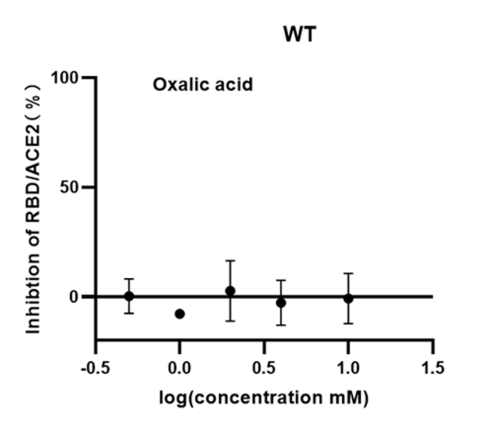


### S2 Fig. Inhibition of positive control on the interaction between ACE2 and SARS-CoV-2 Spike RBD from Delta (B.1.617.2) and Omicron (B.1.1529). Green represents Delta variant and red represents Omicron variant.


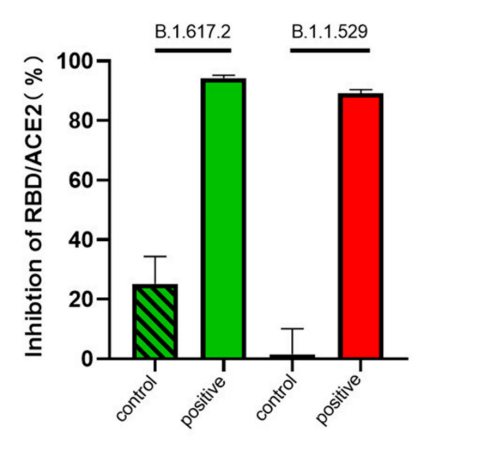

Supplement: S1 Appendix — (DOCX) [file pone.0285722.s001.docx]
