# Supplementary material for: Oxalic acid blocked the binding of spike protein from SARS-CoV-2 Delta (B.1.617.2) and Omicron (B.1.1.529) variants to human angiotensin-converting enzymes 2
Source: PLoS One. 2023 May 18;18(5):e0285722. doi: 10.1371/journal.pone.0285722 (PMC10194883; doi:10.1371/journal.pone.0285722)
Supplement: Attachment — Submitted filename: PONE-D-23-01760_reviewer_.pdf [file pone.0285722.s002.pdf]

PLOS ONE

Oxalic acid blocked the binding of spike protein from SARS-CoV-2 Delta (B.1.617.2) and Omicron (B.1.1.529) variants to human angiotensin-converting enzymes 2

--Manuscript Draft--

**Manuscript Number:**	PONE-D-23-01760
**Article Type:**	Research Article
**Full Title:**	Oxalic acid blocked the binding of spike protein from SARS-CoV-2 Delta (B.1.617.2) and Omicron (B.1.1.529) variants to human angiotensin-converting enzymes 2
**Short Title:**	OA blocked the binding of spike protein from SARS-CoV-2 variants to hACE2
**Corresponding Author:**	Lin Miao<br>Tianjin University of Traditional Chinese Medicine<br>Tianjin, None Selected CHINA
**Keywords:**	oxalic acid; SARS-CoV-2; Delta; Omicron; receptor binding domain; human angiotensin-converting enzymes 2 receptor
**Abstract:**	An epidemic of Corona Virus Disease 2019 (COVID-19) caused by severe acute respiratory syndrome coronavirus 2 (SARS-CoV-2) is spreading worldwide. Moreover, the emergence of SARS-CoV-2 variants of concern, such as Delta and Omicron, has seriously challenged the application of current therapeutics including vaccination and drugs. Relying on interaction of spike protein with receptor angiotensin-converting enzymes 2 (ACE2), SARS-CoV-2 successfully invades to the host cells, which indicates a strategy that identification of small-molecular compounds to block the entry is of great significance for COVID-19 prevention. Our study evaluated the potential efficacy of natural compound oxalic acid (OA) as an inhibitory agent against SARS-CoV-2 invasion, particular on the interaction of the receptor binding domain (RBD) of Delta and Omicron variants to ACE2. By employing a competitive binding assay in vitro, OA significantly blocked the binding of RBDs from Delta B.1.617.2 and Omicron B.1.1.529 to ACE2, but has no effect on the wide-type SARS-CoV-2 strain. Furthermore, OA inhibited the entries of Delta and Omicron pseudovirus into ACE2 high expressing-HEK293T cells. By surface plasmon resonance (SPR) assay, the direct bindings of OA to RBD and ACE2 were analyzed and OA had both affinities with RBDs of B.1.617.2 and B.1.1.529 and with ACE2. Molecular docking predicted the binding sites on the RBD-ACE2 complex and it showed similar binding abilities to both complex of variant Delta or Omicron RBD and ACE2. In conclusion, we provided a promising novel small-molecule compound OA as an antiviral candidate by blocking the cellular entries of SARS-CoV-2 variants.
**Order of Authors:**	Meng Wang
	Huimin Yan
	Lu Chen
	Yu Wang
	Lin Li
	Han Zhang
	Lin Miao
**Additional Information:**	
**Question**	**Response**
**Financial Disclosure**<br><br>Enter a financial disclosure statement that describes the sources of funding for the work included in this submission. Review the submission guidelines for detailed	The funders had no role in study design, data collection and analysis, decision to publish, or preparation of the manuscript.

Powered by Editorial Manager® and ProduXion Manager® from Aries Systems Corporation

requirements. View published research articles from *PLOS ONE* for specific examples.<br><br>This statement is required for submission and **will appear in the published article** if the submission is accepted. Please make sure it is accurate.<br><br>**Unfunded studies**<br>Enter: *The author(s) received no specific funding for this work.*<br><br>**Funded studies**<br>Enter a statement with the following details:<br>• Initials of the authors who received each award<br>• Grant numbers awarded to each author<br>• The full name of each funder<br>• URL of each funder website<br>• Did the sponsors or funders play any role in the study design, data collection and analysis, decision to publish, or preparation of the manuscript?<br>• **NO** - Include this sentence at the end of your statement: *The funders had no role in study design, data collection and analysis, decision to publish, or preparation of the manuscript.*<br>• **YES** - Specify the role(s) played.<br><br>* typeset	
**Competing Interests**<br><br>Use the instructions below to enter a competing interest statement for this submission. On behalf of all authors, disclose any competing interests that could be perceived to bias this work—acknowledging all financial support and any other relevant financial or non-financial competing interests.<br><br>This statement is required for submission and **will appear in the published article** if the submission is accepted. Please make sure it is accurate and that any funding sources listed in your Funding Information later in the submission form are also declared in your Financial Disclosure	The authors have declared that no competing interests exist.

statement.<br><br>View published research articles from *PLOS ONE* for specific examples.<br><br>**NO authors have competing interests**<br><br>Enter: *The authors have declared that no competing interests exist.*<br><br>**Authors with competing interests**<br><br>Enter competing interest details beginning with this statement:<br><br>*I have read the journal's policy and the authors of this manuscript have the following competing interests: [insert competing interests here]*<br><br>* typeset	
**Ethics Statement**<br><br>Enter an ethics statement for this submission. This statement is required if the study involved:<br><br>• Human participants<br>• Human specimens or tissue<br>• Vertebrate animals or cephalopods<br>• Vertebrate embryos or tissues<br>• Field research<br><br>Write "N/A" if the submission does not require an ethics statement.<br><br>General guidance is provided below. Consult the submission guidelines for detailed instructions. **Make sure that all information entered here is included in the Methods section of the manuscript.**	N/A

**Format for specific study types**<br><br>**Human Subject Research (involving human participants and/or tissue)**<br>• Give the name of the institutional review board or ethics committee that approved the study<br>• Include the approval number and/or a statement indicating approval of this research<br>• Indicate the form of consent obtained (written/oral) or the reason that consent was not obtained (e.g. the data were analyzed anonymously)<br><br>**Animal Research (involving vertebrate animals, embryos or tissues)**<br>• Provide the name of the Institutional Animal Care and Use Committee (IACUC) or other relevant ethics board that reviewed the study protocol, and indicate whether they approved this research or granted a formal waiver of ethical approval<br>• Include an approval number if one was obtained<br>• If the study involved *non-human primates*, add *additional details* about animal welfare and steps taken to ameliorate suffering<br>• If anesthesia, euthanasia, or any kind of animal sacrifice is part of the study, include briefly which substances and/or methods were applied<br><br>**Field Research**<br><br>Include the following details if this study involves the collection of plant, animal, or other materials from a natural setting:<br>• Field permit number<br>• Name of the institution or relevant body that granted permission	
**Data Availability**<br><br>Authors are required to make all data underlying the findings described fully available, without restriction, and from the time of publication. PLOS allows rare exceptions to address legal and ethical concerns. See the PLOS Data Policy and FAQ for detailed information.	Yes - all data are fully available without restriction

A Data Availability Statement describing where the data can be found is required at submission. Your answers to this question constitute the Data Availability Statement and **will be published in the article**, if accepted.<br><br>**Important:** Stating 'data available on request from the author' is not sufficient. If your data are only available upon request, select 'No' for the first question and explain your exceptional situation in the text box.<br><br>Do the authors confirm that all data underlying the findings described in their manuscript are fully available without restriction?	
**Describe where the data may be found in full sentences. If you are copying our sample text, replace any instances of XXX with the appropriate details.**<br><br>• If the data are **held or will be held in a public repository**, include URLs, accession numbers or DOIs. If this information will only be available after acceptance, indicate this by ticking the box below. For example: *All XXX files are available from the XXX database (accession number(s) XXX, XXX.*).<br>• If the data are all contained **within the manuscript and/or Supporting Information files**, enter the following: *All relevant data are within the manuscript and its Supporting Information files.*<br>• If neither of these applies but you are able to provide **details of access elsewhere**, with or without limitations, please do so. For example:<br><br>*Data cannot be shared publicly because of [XXX]. Data are available from the XXX Institutional Data Access / Ethics Committee (contact via XXX) for researchers who meet the criteria for access to confidential data.*<br><br>*The data underlying the results presented in the study are available from (include the name of the third party*	All relevant data are within the manuscript and its Supporting Information files.

and contact information or URL).<br>• This text is appropriate if the data are owned by a third party and authors do not have permission to share the data.<br><br>* typeset	
Additional data availability information:	Tick here if the URLs/accession numbers/DOIs will be available only after acceptance of the manuscript for publication so that we can ensure their inclusion before publication.

Powered by Editorial Manager® and ProduXion Manager® from Aries Systems Corporation

Manuscript
Click here to access/download;Manuscript;manuscript.docx

Oxalic acid blocked the binding of spike protein from SARS-CoV-2 Delta (B.1.617.2) and Omicron (B.1.1.529) variants to human angiotensin-converting enzymes 2

Meng Wang[1,2#], Huimin Yan[2,3], Lu Chen[1,2,3], Yu Wang[1,2,3], Lin Li[1,2,3], Han Zhang[1,2,3*], Lin Miao[1,2,3*]

1 State Key Laboratory of Component-based Chinese Medicine, Tianjin University of Traditional Chinese Medicine, Tianjin, 301617, China; Tel: +86-22-59596242; Fax: +86-22-59596586;

2 Institute of Traditional Chinese Medicine, Tianjin University of Traditional Chinese Medicine, Tianjin, 301617, China; Tel: +86-22-59596242; Fax: +86-22-59596586;

3 Key Laboratory of Pharmacology of Traditional Chinese Medical Formulae, Ministry of Education, Tianjin University of Traditional Chinese Medicine, Tianjin, 301617, China; Tel: +86-22-59596171; Fax: +86-22-27386453

Meng Wang, biubiutyuiop@163.com

Huimin Yan, HuiminYan7978@163.com

Lu Chen, chenlutjutcm@tjutcm.edu.cn

Yu Wang, wangyu@tjutcm.edu.cn

Lin Li, 13516122373@126.com

*Correspondence: mmmlin@tjutcm.edu.cn; zhanghan0023@126.com; Tel.: +86-22-59596242

Abstract

An epidemic of Corona Virus Disease 2019 (COVID-19) caused by severe acute respiratory syndrome coronavirus 2 (SARS-CoV-2) is spreading worldwide. Moreover, the emergence of SARS-CoV-2 variants of concern, such as Delta and Omicron, has seriously challenged the application of current therapeutics including vaccination and drugs. Relying on interaction of spike protein with receptor angiotensin-converting enzymes 2 (ACE2), SARS-CoV-2 successfully invades to the host cells, which indicates a strategy that identification of small-molecular compounds to block the entry is of great significance for COVID-19 prevention. Our study evaluated the potential efficacy of natural compound oxalic acid (OA) as an inhibitory agent against SARS-CoV-2 invasion, particular on the interaction of the receptor binding domain (RBD) of Delta and Omicron variants to ACE2. By employing a competitive binding assay *in vitro*, OA significantly blocked the binding of RBDs from Delta B.1.617.2 and Omicron B.1.1.529 to ACE2, but has no effect on the wide-type SARS-CoV-2 strain. Furthermore, OA inhibited the entries of Delta and Omicron pseudovirus into ACE2 high expressing-HEK293T cells. By surface plasmon resonance (SPR) assay, the direct bindings of OA to RBD and ACE2 were analyzed and OA had both affinities with RBDs of B.1.617.2 and B.1.1.529 and with ACE2. Molecular docking predicted the binding sites on the RBD-ACE2 complex and it showed similar binding abilities to both complex of variant Delta or Omicron RBD and ACE2. In

conclusion, we provided a promising novel small-molecule compound OA as an antiviral candidate by blocking the cellular entries of SARS-CoV-2 variants.

Keywords

oxalic acid; SARS-CoV-2; Delta; Omicron; receptor binding domain; human angiotensin-converting enzymes 2 receptor

Introduction

The pandemic of coronavirus disease 2019 (COVID-19) caused by severe acute respiratory disease coronavirus 2 (SARS-CoV-2) has confirmed cases over 500 million with more than 6 million deaths globally by 20 May 2022 [1]. Furthermore, emergence of mutations in SARS-CoV-2 resulted in unexpected disturbs in extremely high infectivity and unexpected ability of immune escape [2-4]. Within distinct variants of concern (VOCs), Delta variant (B.1.617.2) was first reported in India in October 2020 and compared to the wild-type, studies have shown that the Delta variant has nearly 97% increase in the effective reproductive number of virus and thus has more viral loads and higher risks of hospitalization, intensive care unit admission and mortality in infected patients [5-6]. Omicron variant (B.1.1.529) was first identified in Botswana in November 2021. Although evidence showed that Omicron coronavirus causes milder symptoms than previous variants, it still well-concerns that due its high transmissibility, viral infectivity and immune evasion [7-8]. Omicron may be ten times more infectious than the original SARS-CoV-2, and twice as infectious as the Delta variant due to its mutations [9]. Studies have also revealed that the

Omicron variant is partially resistant to the neutralizing activity of therapeutic antibodies and convalescent sera [10-12], which poses significant threat for the clinical effectiveness of the current vaccines and therapeutic antibodies.

SARS-CoV-2 belongs to the β-coronaviruses, which are enveloped, single, and positive-stranded RNA viruses [13]. Its genome DNA encodes the replicase polyprotein and a series of its structural proteins including Spike (S), Envelope, Membrane, and Nucleocapsid proteins [14]. Among these structural proteins, S protein plays the most critical role during viral attachment, fusion and entry into target cells [15]. It consists of two subunit S1 and S2, and within S1 a receptor-binding domain (RBD) is considered as the direct binding site to the host receptor angiotensin-converting enzyme 2 (ACE2), which will be subsequently proteolytically activated by human proteases and finally help virus entry into the cells [16-17]. Although a series of options including anti-viral drugs, vaccines, and monoclonal antibodies have been marketed against SARS-CoV-2, the emergence of viral escape mutants continually calls for the development of drugs and vaccines against variants [18]. Targeting the interaction of S protein with ACE2 receptor has thus been considered as a promising target for drug identification that may contribute to the therapeutic strategy of COVID-19 [19].

Natural products are a good source of molecules with low-toxicity and stable activity for therapeutic development [20]. Oxalic acid (OA) is a strong dicarboxylic acid (Figure 1)

occurring in various plants and vegetables such as sugar beet and spinach. It has been identified as a lipase inhibitor to reduce the absorption of fat by intestinal cells [21]. However, its anti-viral effect has not been reported. In this study, the potential inhibitory effect of OA on prevention of SARS-CoV-2 entries was investigated by targeting at the complex of S protein and ACE2 *in vitro* and in pseudovirus assays. The variant specificities of OA among wild type, Delta B.1.617.2 and Omicron B.1.1529 were also compared, its direct binding to either S or ACE2 were determined and the potential binding sites were further predicted as well. Our results will provide evidence whether OA blocked the binding of SARS-CoV-2 invasion, indicating its potential role in drug discovery against COVID-19.

Results

OA inhibited RBDs of SARS-CoV-2 Delta (B.1.617.2) or Omicron (B.1.1529) bound to ACE2.

OA was tested in vitro for its inhibitory effect on RBD-ACE2 interaction by ELISA. As Fig.2A shown, OA blocked the interaction between Delta RBD and ACE2 in a dose-dependent manner with an IC_{50} of 2.740 mM (Fig. 2A). Similarly, OA also significantly inhibited ACE2 binding to Omicron RBD with an IC_{50} of 1.059 mM (Fig. 2B). In contrast, no inhibitory effect of OA was detected on ACE2 binding to wild type of SARS-CoV-2 RBD (Supplementary Fig.1). Recombinant neutralizing antibody provided within was used as a positive control, and it strongly inhibited the formation of ACE2-RBD complexes (Supplementary Fig.2).

OA suppressed the entries of pseudovirus of SARS-CoV-2 Delta (B.1.617.2) and Omicron (B.1.1529) into ACE2h cells

Before pseudovirus infection assay, cell viability was first determined in response to OA and data showed that no significant cytotoxic effect was detected in ACE2^{h} cells even at 100 μM (Fig. 3). Pseudovirus infection was then performed to detect the inhibitory effect of OA on the viropexis of SARS-CoV-2 Delta and Omicron variants. As shown in Fig. 4, OA inhibited the entry of SARS-CoV-2 Delta pseudovirus into ACE2^{h} cells in a concentration-dependent manner with IC$_{50}$ of 26.04 μM. Consistently, the expression of green fluorescent protein in ACE2^{h} cells was significantly reduced caused by ACE2-Fc or OA in the images photographed by a fluorescence microscope (Fig. 4C).

Similarly, ACE2^{h} cells were infected with SARS-CoV-2 pseudovirus Omicron (B.1.1529), and OA significantly reduced the luciferase activity when compared to the control (Fig. 5A). Photographed images were shown in Fig. 5B under the concentration of 10 μM as well.

OA directly bound to ACE2 and RBDs of SARS-CoV-2 Delta (B.1.617.2) and Omicron (B.1.1529)

To identify the direct interactions of OA on ACE2 and RBD, we further addressed the SPR assay. OA brought about a concentration-dependent resonance change when flowing through the sensor chip coated with either ACE2 (Fig. 6A), RBD Delta (Fig. 6B) or

Omicron (Fig. 6C). The K_D values were then calculated by fitting the affinity data at various concentrations of OA and recorded in Fig. 6D.

The predicted binding sites of OA to the complex of ACE2 with Delta or Omicron RBDs

Recently, it has been well reported that the binding structures of the Delta and Omicron RBDs with ACE2 [18-19], which provide as important targets for candidate screening and site identification. As shown in Figure 7, the active cores were set in Delta RBD-ACE2 (Fig. 7A) or Omicron RBD-ACE2 complex (Fig. 7B). In details, Delta RBD interacted with ACE2 at the residue THR500, GLY502, GLN498, GLY496, GLY446, TYR449, GLN493, LYS417, TYR489, ASN487 and ALA475 with TYR41, LYS353, GLN42, ASP38, LYS31, GLU35, ASP30, TYR83, GLN24 and SER19 in ACE2 via hydrogen bonds, and the residue ASN501, GLY502, TYR505, PHE456, LEU455, TYR453, and PHE486 stabilized with ARG357, ASN330, ASP355, GLY354, GLU37, THR27, HIS34, PHE28, MET82 of ACE2 through non-bonded interactions. The residues involved in the interaction of Omicron RBD and ACE2 included the ARG493, TYR453, TYR501, SER496, GLY502, ASN477, THR500, ARG498 in RBD and GLU35, HIS34, GLY354, LYS353, SER19, TYR41 and GLN42 in ACE2 via hydrogen bonds, as well as the residue PHE456, LEU455, HIS505, TYR489, ALA475, ASN487, GLY476, TYR449 and PHE486 of Omicron RBD with ASP38, THR27, PHE28, TYR83, ARG357, LYS31, ASP355, GLN24, LEU79, MET82 of ACE2 through non-bonded contacts (Fig. 7B).

Next, molecular docking was applied to visually inspect and predict the interfering sites of OA on the complexes of Delta or Omicron RBD with ACE2. As shown in Fig. 8A, OA interacted with the residue LYS353 of ACE2 via the conventional hydrogen bond. A carbon hydrogen bond with Delta RBD residues ARG403 and GLY496 and an attractive charge with residues LYS353 and ARG403 were formed with OA as well.

In Omicron RBD-ACE2, OA bound to the residues LYS353 (Fig. 8B) via the conventional hydrogen bond. It also formed a carbon hydrogen bond with residue HIS34 and ARG403 and an attractive charge with LYS353 and ARG403 residues. The interaction energy scores were estimated as -33.9 and -30.7 kJ/mol. ting a comparable binding affinity of OA interacting with the complex ACE2-RBD Delta and Omicron. The docking scores and the residues interacting with OA were summarized in Table 3.

Discussion

The ongoing COVID-19 pandemic rapidly spread around the world, resulting in enormous consequences in health, economic, and social aspects. With the high mutation rate of the virus and the increased risk of immune viral escape, emerging variants [24-25] exhibited antiviral resistant and threatened the global health care [26-27]. Thus, novel and effective drug are urgently needed for COVID-19 treatment, especially targeted at the early step for infectious prevention. Our study first identified a potential role of natural small molecule OA on blocking the binding of Delta and/or Omicron spike to ACE2. Recently, a report found that a traditional drug cysteamine prescribed to decrease intralysosomal cystine

accumulation [28] exerted direct antiviral actions against SARS-CoV-2 Delta and Omicron variants, in addition to the wild type virus [29]. Using multiple and appropriate screening approaches to extend existed drug applicability as well as identify novel candidate drugs may provide evidence as a future candidate for prevention of SARS-CoV-2 variant infections. However, more cellular and animal assays will be needed to further confirm their inhibitory effects, which are allowed to perform only in a biosafety level-3.

Blocking of the interaction between RBD and ACE2 by inhibitors may be due to the binding to RBD or ACE2, or both. Here, in our study, we first found that OA effectively prevented the binding of the RBD from Delta and omicron variants to the ACE2 receptor by competitive ELISA (Fig. 2), indicating the possibilities of OA on interaction with either RBD or ACE2. In functional assay, pseudovirus infection data confirmed that interfering with ACE2-RBD complex by OA contributes to the SARS-CoV-2 viral entrance to cells (Fig. 4, Fig. 5), as we expected. However, we are still uncertain on the direct interaction of OA with RBD and ACE2. Based on the SPR assay, we further demonstrated that OA directly binding to both RBD and ACE2 (Fig. 6). The K_D values indicated that OA exhibited a higher affinity for ACE2 than those of Delta and Omicron RBDs.

As far as we have known, ACE2 is part of the renin–angiotensin–aldosterone system (RAAS) involved in regulating cardiovascular processes through reduction of blood pressure [30]. It is expressed and located in the cell membranes in several organs such as the lungs, heart, and kidneys. A series of ACE2 inhibitors have been applied in searching for

neutralization of the SARS-CoV-2 virus entries to limit the infection and replication. Here in our study, we found that ACE2 was one of OA's targets (Fig. 6A) to interfere with the interaction between the spike protein and ACE2. However, further investigation regarding the effect of OA on the RAAS is required in the future.

We performed the virtual docking assay to predict the binding structures of OA on RBD-ACE2 binding interfaces of Delta and Omicron. The computed binding conformations appeared with similar poses (Fig.8), and interaction energy scores were comparable as well (Table 3), indicating an equal binding affinity of OA to the complex ACE2-RBD Delta and ACE2-RBD Omicron. We also presented the amino acid residues in ACE2-RBD complexes involved in the interaction with OA interfering, which may be some clues for the design of neutralizing agents for SARS-CoV-2 and further confirmation assay are needed.

OA is an organic compound found in many plants such as leafy greens, vegetables, fruits, cocoa, nuts, and seeds. It is widely used as an acid rinse in laundries, where it is effective in removing rust and ink stains because it converts most insoluble iron compounds into a soluble complex ion. So far, no anti-viral effect of OA has been reported yet. Here, in our study, we first reported that OA has abilities to directly bind Delta (B.1.617.2) and Omicron (B.1.1.529) RBD as well as ACE2, by which it effectively blocked SARS-CoV-2 variant entry to cells and may be considered as an anti-SARS-CoV-2 candidate for COVID-19 drug development.

Conclusion

OA was identified as novel active constituents which blocked both the binding of SARS-CoV-2 Delta (B.1.617.2) and Omicron (B.1.1.529) RBD to ACE2 *in vitro*, suggesting the therapeutic candidates of OA for COVID-19 treatment.

Materials and Methods

Oxalic acid

Oxalic acid was purchased from YuanYe Bio-Technonlogy Co. Shanghai, China with a purity of ≥98% (Cat. No. B20132) and dissolved in dimethyl sulfoxide (DMSO) and stored at-20°C.

Cell culture

ACE2 high expressing-HEK293T cells ($ACE2^h$ cells) were kindly provided by Prof Guo Xiaohuan from Tsinghua University. $ACE2^h$ cells were kept in Dulbecco's Modification of Eagle's Medium (DMEM) (Kibbutz Beit Haemek, Israel, Cat. No. 2110050) containing 10% FBS (Invigentech, CA, US, Cat. No. A6907FBS-500) and 1% penicillin-streptomycin (VivaCell, Shanghai, China, Cat. No.C3421-0100), and cultured at 37 °C containing 5% of CO_2.

Cytotoxicity assay

For cytotoxicity assay, $ACE2^h$ cells were seeded into 96-well plates at a density of 1×10^4 cells per well and then treated with different concentrations of OA (0.01, 0.1, 1, 10, 100 μM) for 24 h, then 10 μL of Cell Counting Kit (IV08-500, Invigentech, USA) solution

was added to each well followed by 2 h of incubation. The relative cell viability was assessed by the detection of the absorbance at 450 nm using a microplate reader (Infinite F50, Thermo Fisher, China). The survival rate of ACE2^h cells was calculated as the following formula: Survival rate = $[(OD_{Treated} - OD_{Blank}) / (OD_{Control} - OD_{Blank})] \times 100\%$.

Enzyme-linked immunosorbent assay

According to the instruction (ACROBiosystems, Cat. No. EP111/EP-115, Beijing, China), various concentrations of OA were added to the wells in an ACE2 pre-coated plate followed by addition of HRP-SARS-CoV-2 Spike RBD. After incubation at 37 °C for 1h, wells were washed and the substrate was then added. After incubation at 37℃ for 20 min, the reaction was finally terminated by the addition of stop solution. The absorbance at 450 nm was measured immediately by the microtiter plate reader (Infinite F50, Thermo Fisher, China). The neutralizing antibody provided by ACROBiosystems was used as the positive control. The inhibition rate was calculated as the following formula: Inhibition rate = (1-OD Sample/ OD Negative Control) × 100%.

Pseudovirus entry assay

Pseudovirus from SARS-CoV-2 B.1.617.2 (Cat. No. FNV3718) and B.1.1529 (Cat. No. FNV4122) were purchased from FUBIO (Jiangsu, China). Pseudovirus (2×107 TFU/ml) were pre-incubated with different concentration of OA or ACE2-Fc (GenScript, Cat. No. Z03516, Nanjing, China) as a positive control in a 96-well plate for 1 h at room temperature. ACE2^h cells were then seeded at 3×10^4/well into the plate with the pseudovirus-OA mixture

and incubated for additional 6 h. Then the medium was replaced with fresh medium and incubated for another 48 h. The GFP images were captured by BioTek Lionheart FX Automated Microscope (Agilent Technologies, USA). Cells were harvested and the luciferase activity was measured using the luciferase reagent (Promega, Shanghai, China, Cat. No. G7941) by the microtiter plate reader (PerkinElmer, Victor XLight). The inhibition rate was calculated as the following formula: Inhibition rate = (1- Signal of positive control - Blank control/Signal of Negative control - Blank control) × 100%.

Surface plasmon resonance

Surface plasmon resonance (SPR) measurement was performed at 25 °C using a BIAcore T200 instrument. RBDs of Delta B.1.617.2 (Sino Biological, Beijing, China, Cat. No.40592-V08H90, 50 μg/ml) and Omicron B.1.1.529 (GenScrip, Nanjing, China, Cat. No. Z03728, 50 μg/ml) or ACE2 (Sino Biological Inc., China. Cat. No. 10108-H05H, 102 μg/ml) protein were immobilized on a CM5 sensor chip (Cytiva, Uppsaia, Sweden, Cat. No. 29104988) respectively, and a blank channel was employed as a negative control for each assay. Different concentrations of OA were then injected into the immobilized biosensor chips. The contact time was 120s and dissociation time was 180s. The 1:1 binding model was used to assess the binding kinetics. KD values were calculated with a kinetics model by BIAcore T200 analysis software.

Molecular Docking

Ligand preparation

3D structure of OA was obtained and downloaded from the Chemical library ChemSpider (http://www.chemspider.com/) and converted to PDB files. “Prepare ligands” module in Discovery Studio (DS) 2020 was applied with the candidates followed by “minimize ligands” module.

Protein preparation

High-resolution X-ray crystal structures of S-ACE2 complexes (PDB ID: 7V8B and 7T9L) were taken as targets. To optimize the structure, the complex was initially processed by “prepare protein” module in DS 2020, followed by supplementation of the missing sidechains, removement of the water molecules and protonation suitable at pH 7.4. According to the previous findings [22-23], the active cores of RBD bound to ACE2 were set in Table1 and Table2.

Docking

The molecular docking of OA was performed at the interface of ACE2-RBD through the semi-flexible docking by CDOCKER module in DS 2020. The computed binding conformation and interaction energy scores were recorded.

Statistical Analysis

Statistical analyses were performed using GraphPad Prism software 8.0. One-way ANOVA was used to determine the statistical significance between different groups (*$p < 0.05$, **$p < 0.01$, and ***$p < 0.001$).

Acknowledgements

The authors would like to extend their sincere appreciation to the National Key Research and Development Project of China (No.2021YFC1712904), National Natural Science Foundation of China (General Program No.82074105) and Research cooperation Project of Guangzhou Xin Huangpu Joint Innovation institute of Chinese Medicine (No.2022OTH009). The authors would like to acknowledge the support of Tsinghua University for providing the ACE2 high expressing-HEK293T cells.

Author contribution

Lin Miao and Han Zhang initiated and designed the study; Meng Wang completed the experimental research under the guidance and supervision of Lin Miao and Han Zhang; Huimin Yan and Meng Wang analysed the data; Lin Miao and Meng Wang wrote the manuscript and drew the figures; Lu Chen, Yu Wang and Lin Li provided assistance for the statistical analysis. All authors read and approved the final manuscript.

Conflicts of interest

The authors declare that they have no known competing financial interests or personal relationships that could have appeared to influence the work reported in this paper.

References

1. WHO. WHO Coronavirus (COVID-19) Dashboard. Geneva: WHO (2022). Available online at: https://covid19.who.int/ (accessed May 19, 2022).
2. Escalera A, Gonzalez-Reiche AS, Aslam S, Mena I, Laporte M, Pearl RL, Fossati A, Rathnasinghe R, Alshammary H, van de Guchte A, Farrugia K, Qin Y, Bouhaddou M, Kehrer T, Zuliani-Alvarez L, Meekins DA, Balaraman V, McDowell C, Richt JA, Bajic G, Sordillo EM, Dejosez M, Zwaka TP, Krogan NJ, Simon V, Albrecht RA, van Bakel H, García-Sastre A, Aydillo T. (2022) Mutations in SARS-CoV-2 variants of concern link to increased spike cleavage and virus transmission. Cell Host Microbe. 30(3):373-387.e7. DOI: 10.1016/j.chom.2022.01.006
3. Tao K, Tzou PL, Nouhin J, Gupta RK, de Oliveira T, Kosakovsky Pond SL, Fera D, Shafer RW. (2021) The biological and clinical significance of emerging SARS-CoV-2 variants. Nat Rev Genet. 22(12):757-773. DOI: 10.1038/s41576-021-00408-x
4. Harvey WT, Carabelli AM, Jackson B, Gupta RK, Thomson EC, Harrison EM, Ludden C, Reeve R, Rambaut A. (2021) COVID-19 Genomics UK (COG-UK) Consortium, Peacock SJ, Robertson DL. SARS-CoV-2 variants, spike mutations and immune escape. Nat Rev Microbiol. 19(7):409-424. DOI: 10.1038/s41579-021-00573-0
5. Sheikh A, McMenamin J, Taylor B, Robertson C. (2021) Public Health Scotland and the EAVE II Collaborators. SARS-CoV-2 Delta VOC in Scotland: demographics, risk

of hospital admission, and vaccine effectiveness. Lancet. 397(10293):2461-2462. DOI: 10.1016/S0140-6736(21)01358-1.

6. Tian D, Sun Y, Zhou J, Ye Q. (2021) The Global Epidemic of the SARS-CoV-2 Delta Variant, Key Spike Mutations and Immune Escape. Front Immunol. 12: 751778. DOI: 10.3389/fimmu.2021.751778.

7. Tian D, Sun Y, Xu H, Ye Q. (2022) The emergence and epidemic characteristics of the highly mutated SARS-CoV-2 Omicron variant. J Med Virol. 94(6):2376-2383. DOI: 10.1002/jmv.27643.

8. Callaway E. (2021) Heavily mutated Omicron variant puts scientists on alert. Nature. 600(7887):21. DOI: 10.1038/d41586-021-03552-w.

9. Liu Y, Rocklöv J. (2022) The effective reproductive number of the Omicron variant of SARS-CoV-2 is several times relative to Delta. J Travel Med. 29(3): taac037. DOI: 10.1093/jtm/taac037.

10. Planas D, Saunders N, Maes P, Guivel-Benhassine F, Planchais C, Buchrieser J, Bolland WH, Porrot F, Staropoli I, Lemoine F, Péré H, Veyer D, Puech J, Rodary J, Baele G, Dellicour S, Raymenants J, Gorissen S, Geenen C, Vanmechelen B, Wawina-Bokalanga T, Martí-Carreras J, Cuypers L, Sève A, Hocqueloux L, Prazuck T, Rey FA, Simon-Loriere E, Bruel T, Mouquet H, André E, Schwartz O. (2022) Considerable escape of SARS-CoV-2 Omicron to antibody neutralization. Nature. 602(7898):671-675. DOI: 10.1038/s41586-021-04389-z.

11. Cao Y, Wang J, Jian F, Xiao T, Song W, Yisimayi A, Huang W, Li Q, Wang P, An R, Wang J, Wang Y, Niu X, Yang S, Liang H, Sun H, Li T, Yu Y, Cui Q, Liu S, Yang X, Du S, Zhang Z, Hao X, Shao F, Jin R, Wang X, Xiao J, Wang Y, Xie XS. (2022) Omicron escapes the majority of existing SARS-CoV-2 neutralizing antibodies. Nature. 602(7898):657-663. DOI: 10.1038/s41586-021-04385-3.
12. Carreño JM, Alshammary H, Tcheou J, Singh G, Raskin AJ, Kawabata H, Sominsky LA, Clark JJ, Adelsberg DC, Bielak DA, Gonzalez-Reiche AS, Dambrauskas N, Vigdorovich V; PSP-PARIS Study Group, Srivastava K, Sather DN, Sordillo EM, Bajic G, van Bakel H, Simon V, Krammer F. (2022) Activity of convalescent and vaccine serum against SARS-CoV-2 Omicron. Nature. 602(7898):682-688. DOI: 10.1038/s41586-022-04399-5.
13. Fung TS, Liu DX. (2022) Similarities and Dissimilarities of COVID-19 and Other Coronavirus Diseases. Annu Rev Microbiol. 75:19-47. DOI: 10.1146/annurev-micro-110520-023212.
14. Hu B, Guo H, Zhou P, Shi ZL. Characteristics of SARS-CoV-2 and COVID-19. (2021) Nat Rev Microbiol. 19(3):141-154. DOI: 10.1038/s41579-020-00459-7.
15. Watanabe Y, Allen JD, Wrapp D, McLellan JS, Crispin M. (2020) Site-specific glycan analysis of the SARS-CoV-2 spike. Science. 369(6501):330-333. DOI: 10.1126/science.abb9983.

16. Wang Q, Zhang Y, Wu L, Niu S, Song C, Zhang Z, Lu G, Qiao C, Hu Y, Yuen KY, Wang Q, Zhou H, Yan J, Qi J. (2020) Structural and Functional Basis of SARS-CoV-2 Entry by Using Human ACE2. Cell. 181(4):894-904.e9. DOI: 10.1016/j.cell.2020.03.045.

17. Lan J, Ge J, Yu J, Shan S, Zhou H, Fan S, Zhang Q, Shi X, Wang Q, Zhang L, Wang X. (2020) Structure of the SARS-CoV-2 spike receptor-binding domain bound to the ACE2 receptor. Nature. 581(7807):215-220. DOI: 10.1038/s41586-020-2180-5.

18. Cox M, Peacock TP, Harvey WT, Hughes J, Wright DW; COVID-19 Genomics UK (COG-UK) Consortium, Willett BJ, Thomson E, Gupta RK, Peacock SJ, Robertson DL, Carabelli AM. (2022) SARS-CoV-2 variant evasion of monoclonal antibodies based on in vitro studies. Nat Rev Microbiol. 28:1–13. DOI: 10.1038/s41579-022-00809-7.

19. Li J, Zhan P, Liu X. (2020) Targeting the entry step of SARS-CoV-2: a promising therapeutic approach. Signal Transduct Target Ther. 5(1):98. DOI: 10.1038/s41392-020-0195-x.

20. Ma LL, Liu HM, Liu XM, Yuan XY, Xu C, Wang F, Lin JZ, Xu RC, Zhang DK. (2021) Screening S protein - ACE2 blockers from natural products: Strategies and advances in the discovery of potential inhibitors of COVID-19. Eur J Med Chem. 226: 113857. DOI: 10.1016/j.ejmech.2021.113857.

21. Liu TT, He XR, Xu RX, Wu XB, Qi YX, Huang JZ, Chen QH, Chen QX. (2018) Inhibitory mechanism and molecular analysis of furoic acid and oxalic acid on lipase. Int J Biol Macromol. 120(Pt B):1925-1934. DOI: 10.1016/j.ijbiomac.2018.09.150.

22. Celik I, Yadav R, Duzgun Z, Albogami S, El-Shehawi AM, Fatimawali, Idroes R, Tallei TE, Emran TB. (2021) Interactions of the Receptor Binding Domain of SARS-CoV-2 Variants with hACE2: Insights from Molecular Docking Analysis and Molecular Dynamic Simulation. Biology (Basel). 10(9):880. DOI: 10.3390/biology10090880.

23. Bahadur Gurung A, Ajmal Ali M, Elshikh MS, Aref I, Amina M, Lee J. (2022) An *in silico* approach unveils the potential of antiviral compounds in preclinical and clinical trials as SARS-CoV-2 omicron inhibitors. Saudi J Biol Sci. 29(6):103297. DOI: 10.1016/j.sjbs.2022.103297.

24. Padhi, A. K., Shukla, R., Saudagar, P., & Tripathi, T. (2020) High-throughput rational design of the remdesivir binding site in the RdRp of SARS-CoV-2: implications for potential resistance. iScience, *24*(1), 101992. DOI: 10.1016/j.isci.2020.101992.

25. Guo K, Barrett BS, Morrison JH, Mickens KL, Vladar EK, Hasenkrug KJ, Poeschla EM, Santiago ML. (2022) Interferon resistance of emerging SARS-CoV-2 variants. Proc Natl Acad Sci U S A. 119(32): e2203760119. DOI: 10.1073/pnas.2203760119.

26. Gobeil SM, Henderson R, Stalls V, Janowska K, Huang X, May A, Speakman M, Beaudoin E, Manne K, Li D, Parks R, Barr M, Deyton M, Martin M, Mansouri K,

Edwards RJ, Eaton A, Montefiori DC, Sempowski GD, Saunders KO, Wiehe K, Williams W, Korber B, Haynes BF, Acharya P. (2022) Structural diversity of the SARS-CoV-2 Omicron spike. Mol Cell. 82(11):2050-2068.e6. DOI: 10.1016/j.molcel.2022.03.028.

27. Iuliano AD, Brunkard JM, Boehmer TK, Peterson E, Adjei S, Binder AM, Cobb S, Graff P, Hidalgo P, Panaggio MJ, Rainey JJ, Rao P, Soetebier K, Wacaster S, Ai C, Gupta V, Molinari NM, Ritchey MD. (2022) Trends in Disease Severity and Health Care Utilization During the Early Omicron Variant Period Compared with Previous SARS-CoV-2 High Transmission Periods - United States, December 2020-January 2022. MMWR Morb Mortal Wkly Rep. 71(4):146-152. DOI: 10.15585/mmwr.mm7104e4.

28. Besouw M, Masereeuw R, van den Heuvel L, Levtchenko E. (2013) Cysteamine: an old drug with new potential. Drug Discov Today. 18(15-16):785-92. DOI: 10.1016/j.drudis.2013.02.003.

29. Alonzi T, Aiello A, Repele F, Falasca L, Francalancia M, Garbuglia AR, Delogu G, Nicastri E, Piacentini M, Goletti D. (2022) Cysteamine exerts in vitro antiviral activity against the SARS-CoV-2 Delta and Omicron variants. Cell Death Discov. 8(1):288. DOI: 10.1038/s41420-022-01080-8.

30. Zisman LS, Keller RS, Weaver B, Lin Q, Speth R, Bristow MR, Canver CC. (2003) Increased angiotensin-(1-7)-forming activity in failing human heart ventricles:

evidence for upregulation of the angiotensin-converting enzyme Homologue ACE2. Circulation. 108(14):1707-12. DOI: 10.1161/01.CIR.0000094734.67990.99.

Figure 1 Chemical structure of oxalic acid

Figure 2 Effects of OA on the interaction between ACE2 and SARS-CoV-2 Spike RBD from Delta (B.1.617.2) and Omicron (B.1.1529). Representative inhibitory curves of ACE2 binding to SARS-CoV-2 RBDs of Delta RBD (A), Omicron RBD (B) in the presence of OA determined by ELISA.

Figure 3 Effect of OA on ACE2^h cell viability. ACE2^h cells were pretreated with different doses of OA and incubated for 24 h. Viability of ACE2^h cells was detected at OD_{450nm}. The proliferation ration was calculated. Data are presented as mean ± *SD*.

Figure 4 The effect of OA on the entry of SARS-COV-2 Delta (B.1.617.2) pseudovirus into ACE2^h cells. (A) The entrance of SARS-CoV-2 Delta pseudovirus into ACE2^h cells was evaluated by the luciferase activity after treated with ACE2-Fc and different concentrations of OA. (B) Inhibition rate was calculated and showed in curves. (C) Representative images were photographed at × 4 magnification using the fluorescence microscope. Data were presented as mean ± S.D. $^{**}p < 0.01$, $^{***}p < 0.001$, compared with control.

Figure 5 The effect of OA on the entry of SARS-COV-2 Omicron (B.1.1.529) pseudovirus into ACE2^h cells. (A) The entrance of SARS-CoV-2 Delta pseudovirus into ACE2^h cells was evaluated by the luciferase activity after treated with ACE2-Fc and different concentrations of OA. (B) Representative images were photographed at × 4

magnification using the fluorescence microscope. Data are presented as mean ± S.D. *p <0.05, **p < 0.01, ***p < 0.001, compared with control.

Figure 6 The binding characters of OA on ACE2 and RBDs of Delta and Omicron. The SPR titration curves of OA interacted with ACE2 protein (A), Delta RBD protein (B) or Omicron RBD protein (C) were shown. The affinity constants of OA to Delta, Omicron or ACE2 protein were calculated and presented (D).

Figure 7 SARS-CoV-2 Delta or Omicron spike protein in complex with human ACE2. Delta (A) and Omicron (B) RBD binding to ACE2 interface were marked.

Figure 8 Virtual docking of OA with RBD-ACE2 binding interfaces. The predicted binding sites of OA to the complex of ACE2 with Delta (A) or Omicron (B) RBD were shown.

Table 1 The active cores of ACE2 and Delta spike protein complexes

	RBD (Delta)	ACE2	Category	Types
	THR500	TYR41	Hydrogen Bond	Conventional Hydrogen Bond
	GLY502			Conventional Hydrogen Bond
	GLN498	LYS353	Hydrogen Bond	Conventional Hydrogen Bond
	GLY496			Conventional Hydrogen Bond
	GLY446	GLN42	Hydrogen Bond	Conventional Hydrogen Bond
	TYR449	ASP38	Hydrogen Bond	Conventional Hydrogen Bond
Site	GLN493	LYS31	Hydrogen Bond	Conventional Hydrogen Bond
		GLU35	Hydrogen Bond	
	LYS417	ASP30	Hydrogen Bond	Salt Bridge
	TYR489	TYR83	Hydrogen Bond	Conventional Hydrogen Bond
	ASN487	GLN24	Hydrogen Bond	Conventional Hydrogen Bond

	ALA475	SER19	Hydrogen Bond	Conventional Hydrogen Bond

Table 2 The active cores of ACE2 and Omicron spike protein complexes

	RBD (Omicron)	ACE2	Category	Types
Site	ARG493	Glu35	Hydrogen Bond	Salt Bridge
Site	TYR453	HIS34	Hydrogen Bond	Conventional Hydrogen Bond
Site	TYR501	HIS34	Hydrogen Bond	Conventional Hydrogen Bond
Site	ASN477	GLY354	Hydrogen Bond	Conventional Hydrogen Bond
Site	ASN477	SER19	Hydrogen Bond	Conventional Hydrogen Bond
Site	SER496	LYS353	Hydrogen Bond	Conventional Hydrogen Bond
Site	GLY502	LYS353	Hydrogen Bond	Conventional Hydrogen Bond
Site	THR500	TYR41	Hydrogen Bond	Carbon Hydrogen Bond
Site	ARG498	GLN42	Hydrogen Bond	Conventional Hydrogen Bond

Click here to access/download;Figure;figure 1.tif

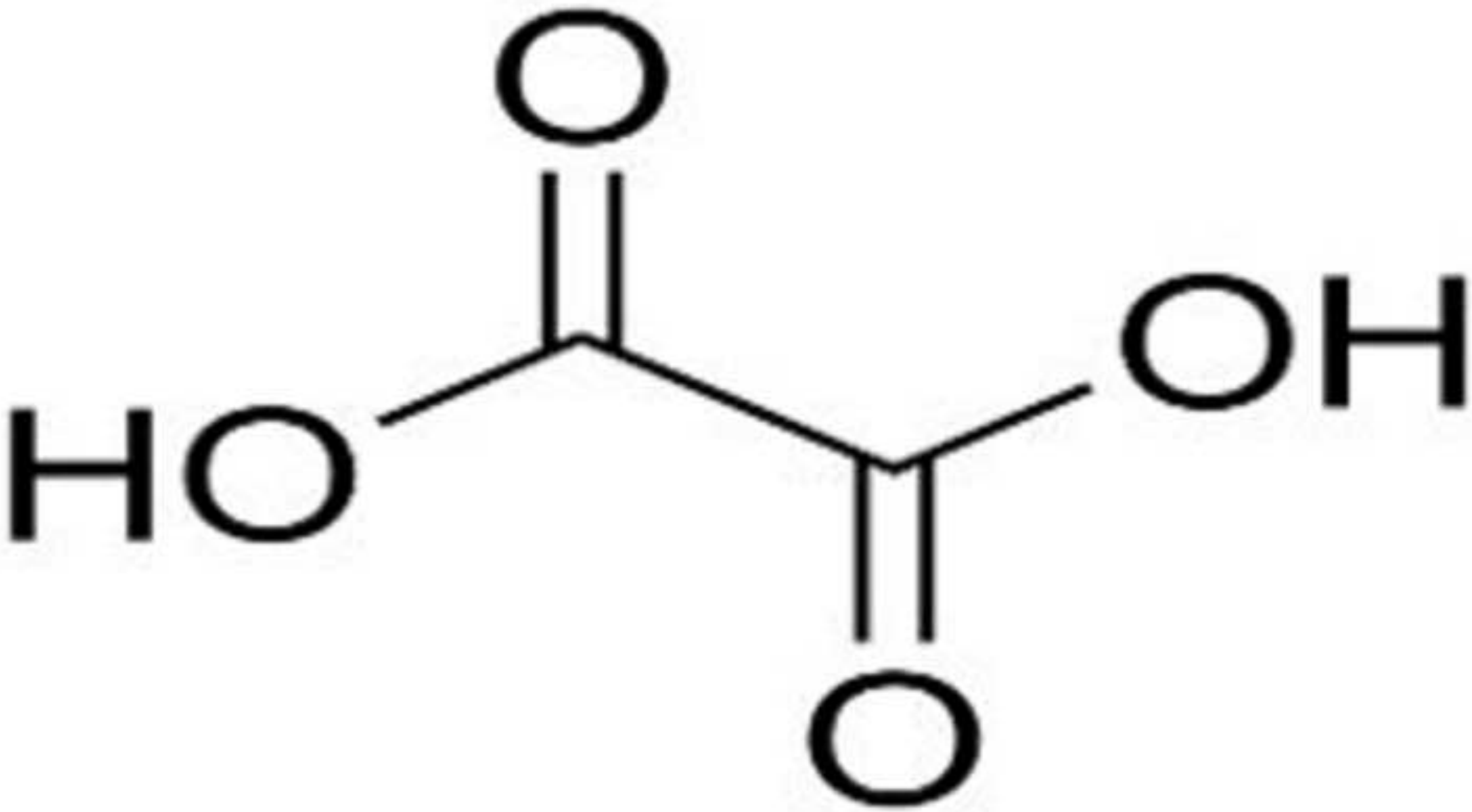

Click here to access/download;Figure;figure 2.tif

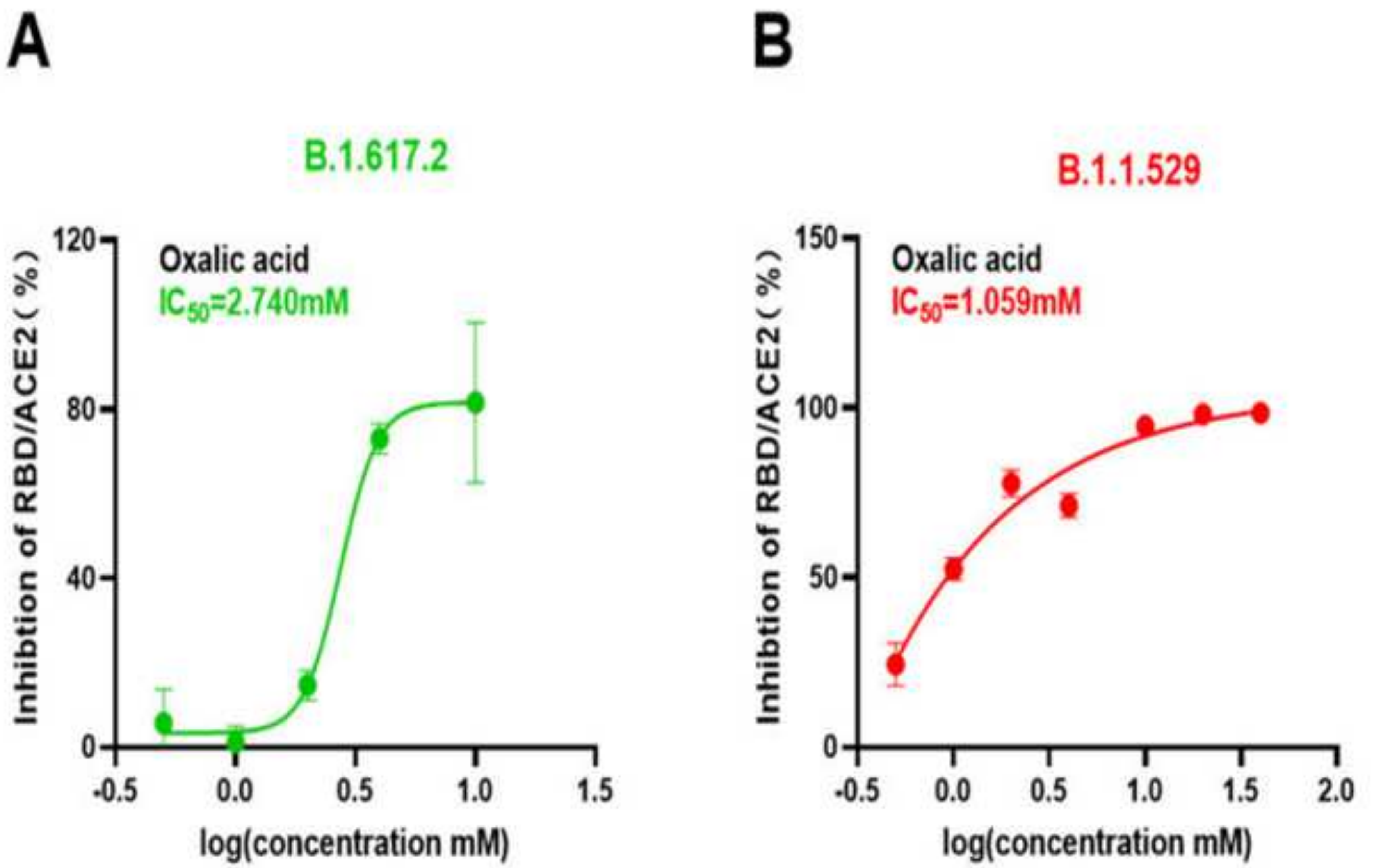

Figure
Click here to access/download;Figure;figure 3.tif

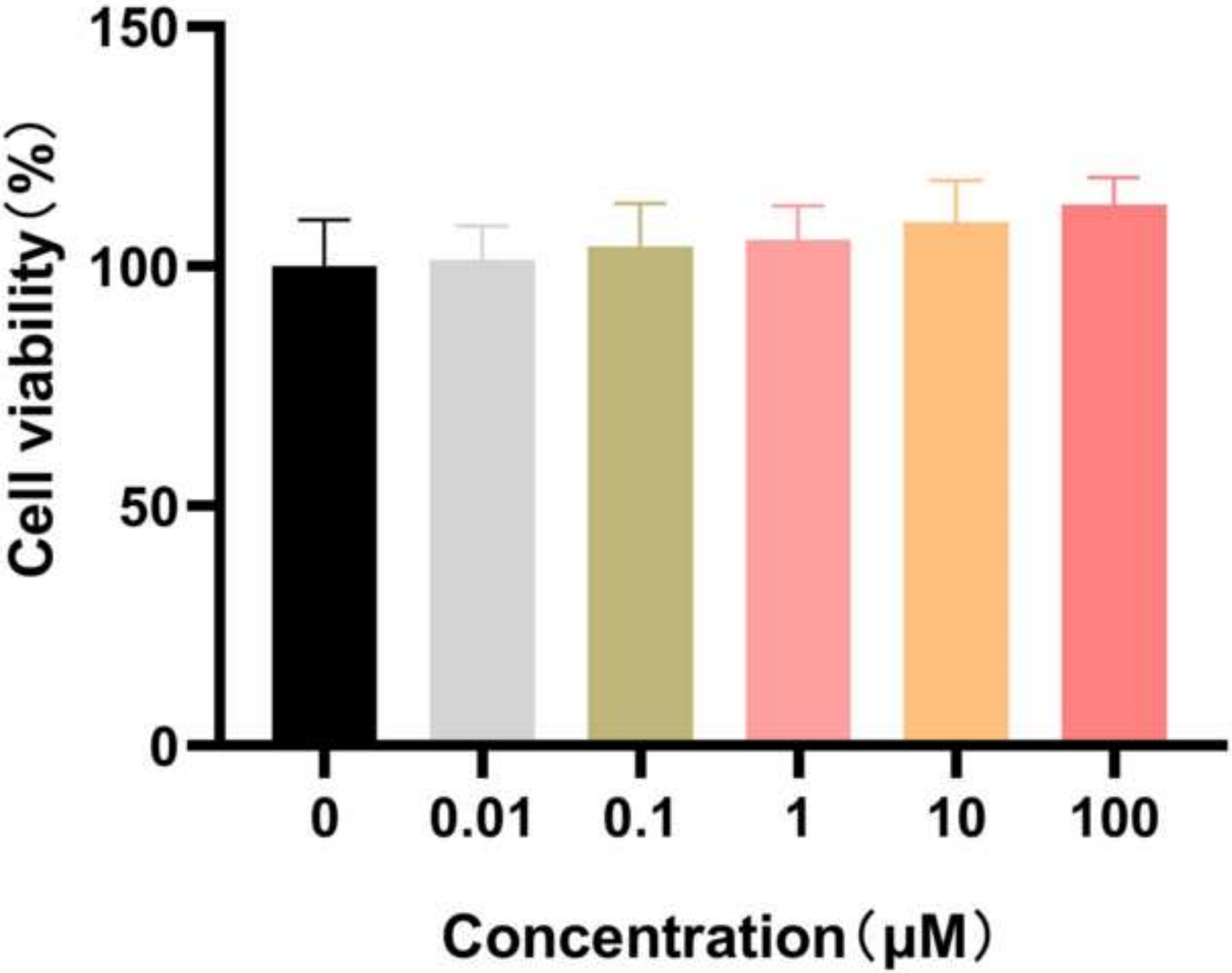

 Click here to access/download;Figure;figure 4.tif

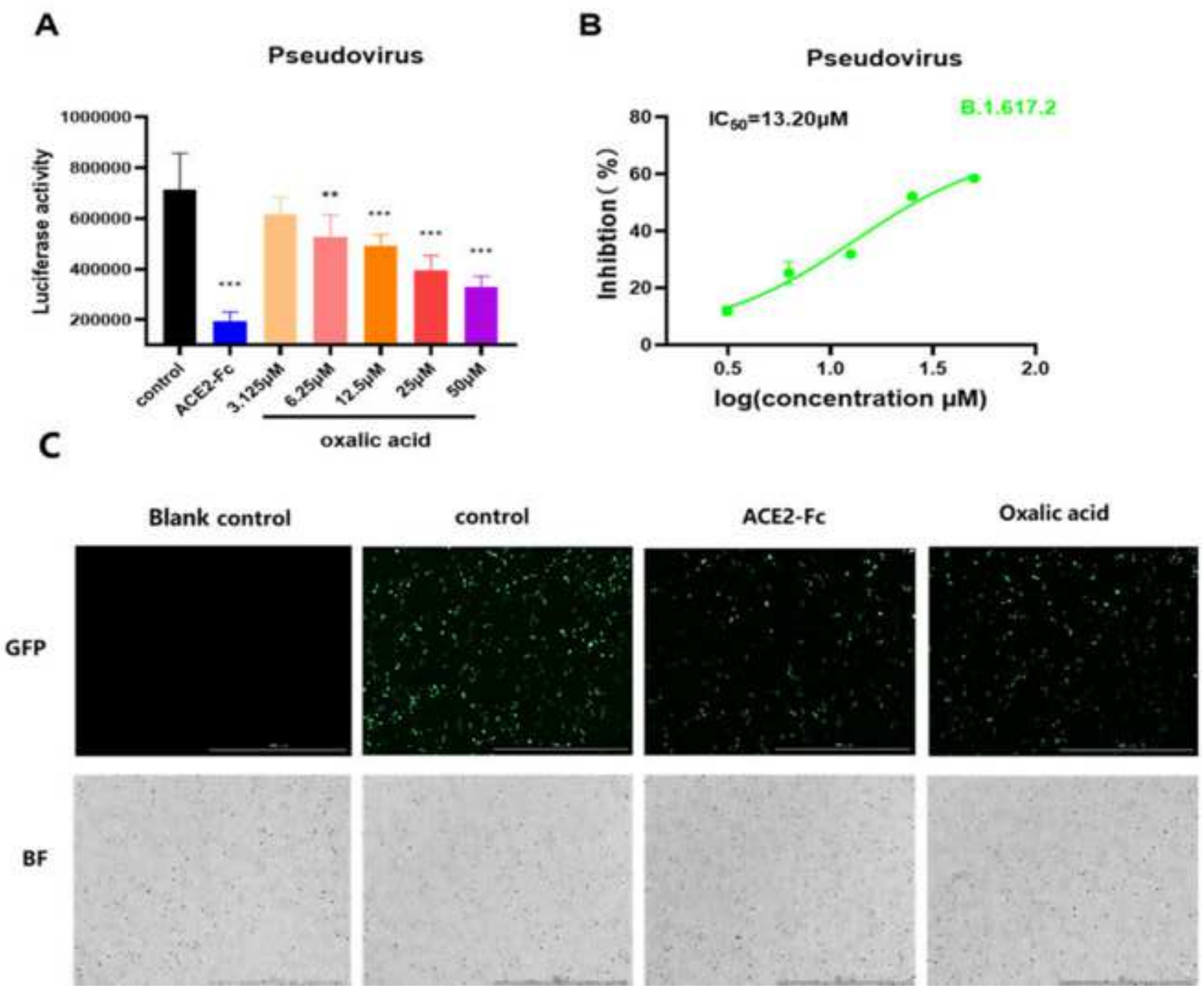

Figure
Click here to access/download;Figure;figure 5.tif

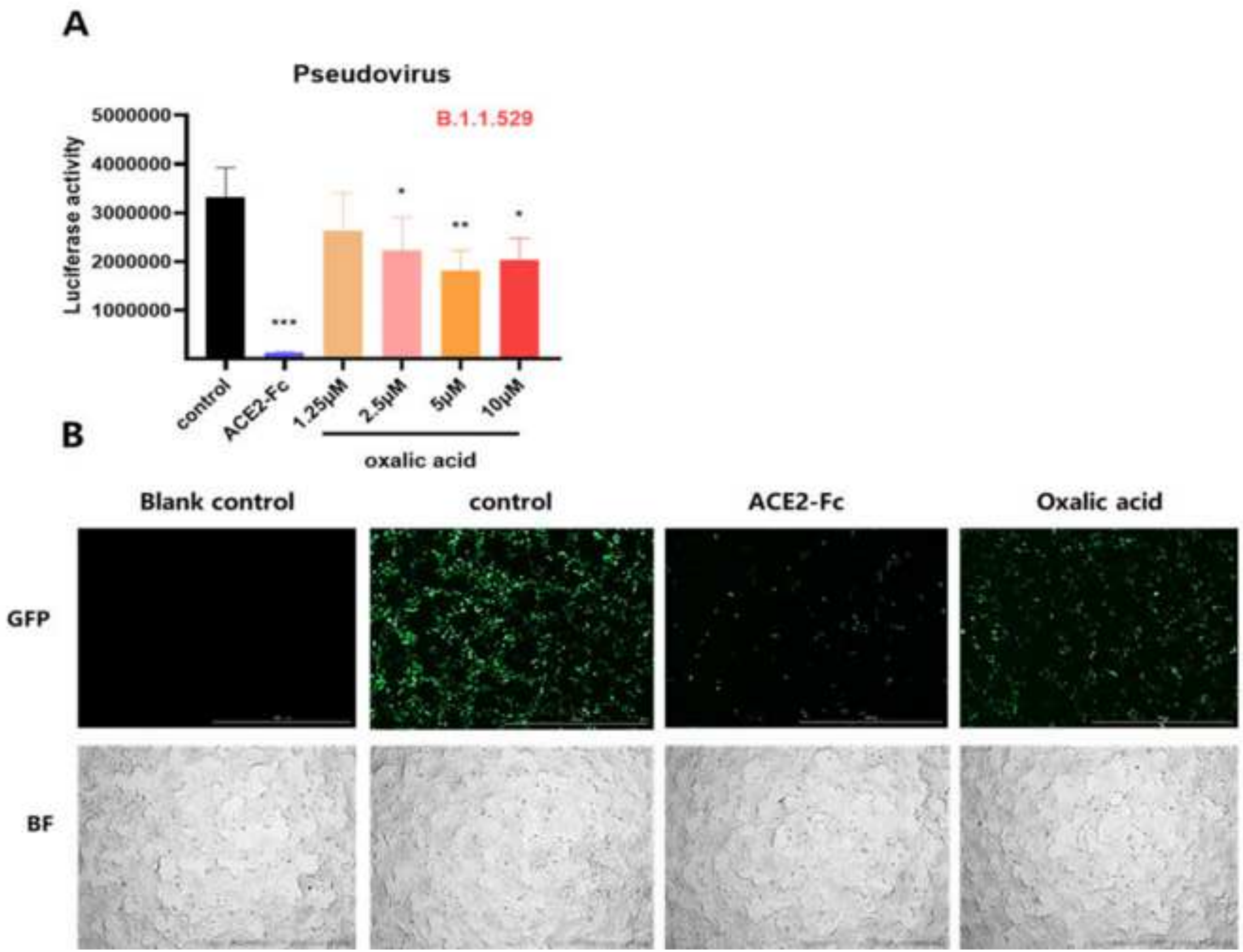

 Click here to access/download;Figure;figure 6.tif

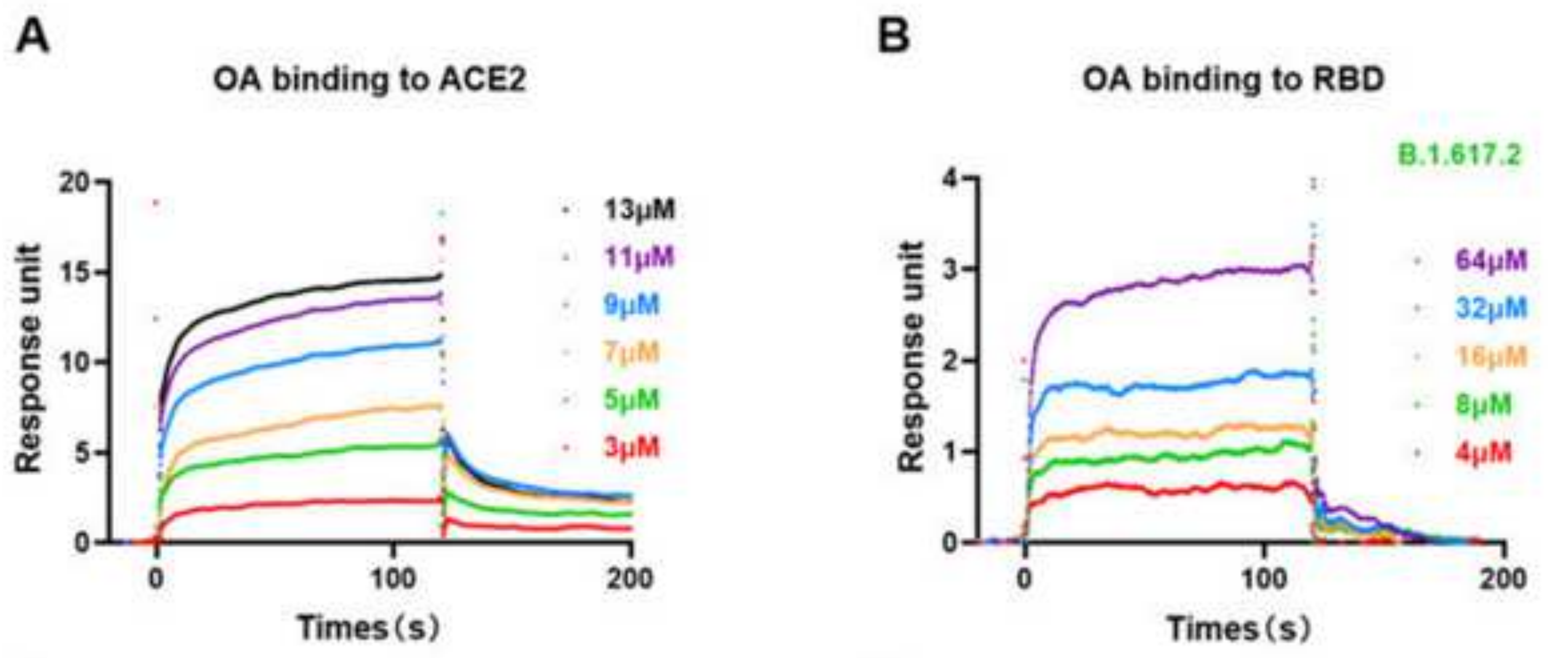

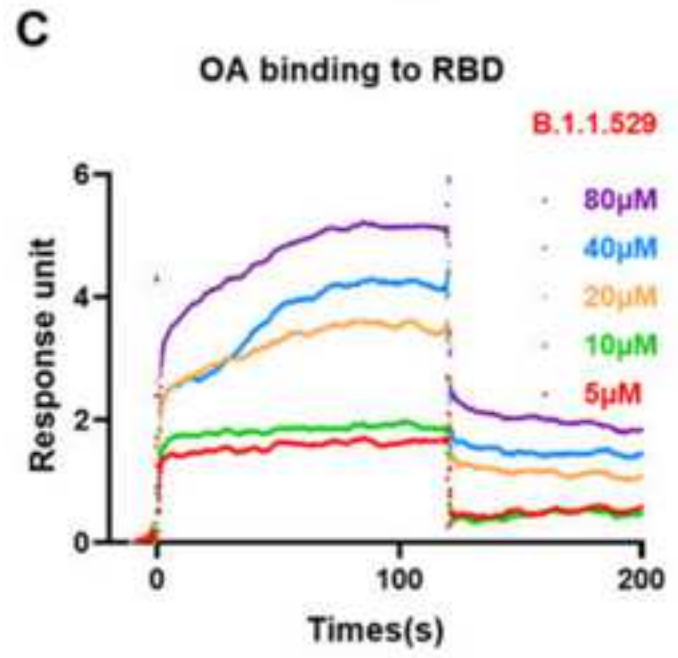

D

Compound	Target	K_D (μM)
Oxalic acid	ACE2	9.66
	S-RBD (B.1.617.2)	25.48
	S-RBD (B.1.1.529)	27.44

Figure
Click here to access/download;Figure;figure 7.tif

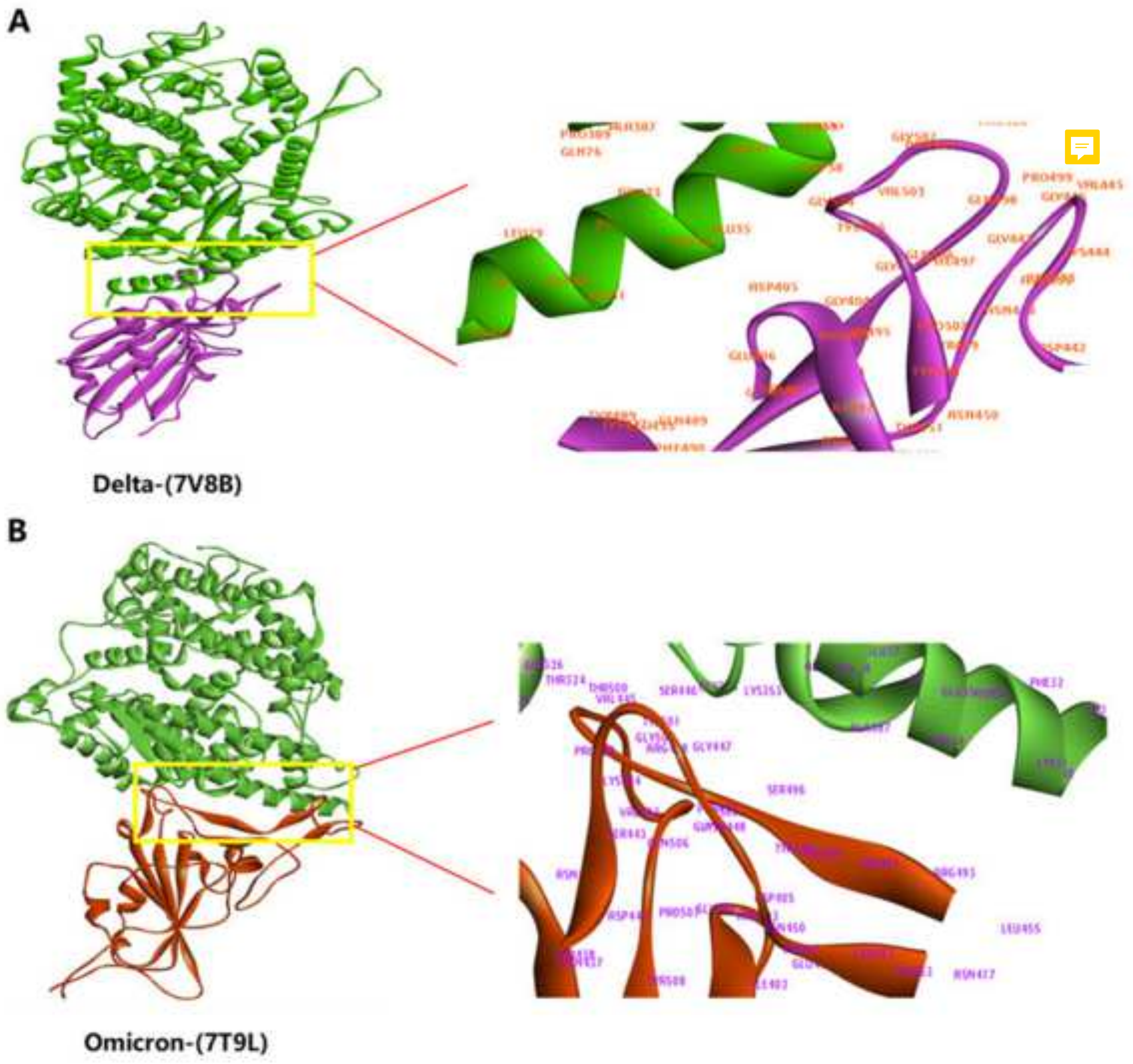

Figure
Click here to access/download;Figure;figure 8.tif

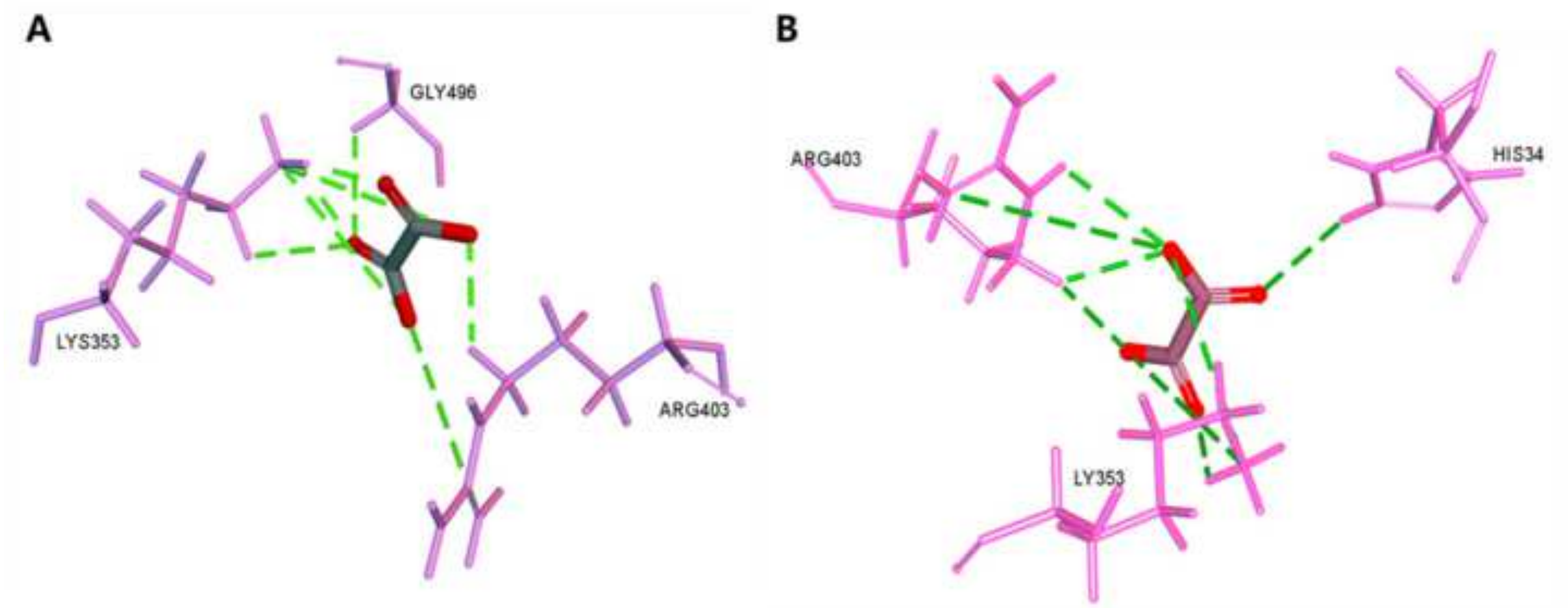

Click here to access/download
Supporting Information
Supplementary materials.docx
