# Supplementary material for: Oxalic acid blocked the binding of spike protein from SARS-CoV-2 Delta (B.1.617.2) and Omicron (B.1.1.529) variants to human angiotensin-converting enzymes 2
Source: PLoS One. 2023 May 18;18(5):e0285722. doi: 10.1371/journal.pone.0285722 (PMC10194883; doi:10.1371/journal.pone.0285722)
Supplement: Attachment — Submitted filename: Response to reviewers.docx [file pone.0285722.s003.docx]

1. line78：formula could be given

**Response: Thank you for reminding of us. The original file was in low format. We have re-uploaded the formula as Fig 1 in the revised manuscript. Please check.**

2. line81：the relation of OA and covid-19 could be added as in the paper:
   Shi D, Yan R, Lv L, Jiang H, Lu Y, Sheng J, Xie J, Wu W, Xia J, Xu K, Gu S, Chen Y, Huang C, Guo J, Du Y, Li L. The serum metabolome of COVID-19 patients is distinctive and predictive. Metabolism. 2021 May; 118:154739. doi: 10.1016/j.metabol.2021.154739. Epub 2021 Mar 2. PMID: 33662365; PMCID: PMC7920809.

**Response: Thank you very much for the literature you provided, which help us find the relationship between COVID-19 and oxalic acid. We have supplemented its findings of OA reported in the paper and labeled them in the *introduction* part started from Line 81 to 84.**

3. Line95：Fig. 2A

**Response: Thank you for your reminding. After your suggestion, we have checked the original data and found one of the values in OA in 100 mM group has been mis-inputted before analysis. We have re-corrected it and replaced the statistical analysis. The refreshed figure was presented in Fig 2A in the revised version.**

4. Line113：Fig.5 inhibition curve

**Response: Thank you for your question. Actually, we performed the pseudovirus** [**luciferase**](https://www.sciencedirect.com/topics/medicine-and-dentistry/luciferase) **assay with different doses of OA in a range from 1.25 to 80μM. However, the inhibitory curve did not be well-fitted (as****below). Therefore, we did not show a curve but the histogram instead.**

5. Line122：Fig. 6D stronger binding to ACE2 should be discussioned

**Response: Thank you for the constructive comment. Following your suggestion, we have made corresponding discussion in the *Discussion* section started from Line 179 to 183.**

6. Line127：not clear how the binding site was determined（using which tool）and side chains are missing in the figures.
(1) **Response: Thank you for the question. We are sorry not to illustrate clearly in the previous manuscript. Here, we supplemented the setting condition and the reference in the Molecular Docking of Material part. Briefly, the active site definition of Delta RBD to ACE2 was determined based on the following publications Han P et al. *Cell* 2022 and Celik I et al. *Biology* 2021 *(See the reference list below). We* summarized the detail residues involved in Table 1 in the revised version and also presented the reference together in the Table 1 below. Similarly, the binding sites of Omicron RBD to ACE2 was selected based on the following publications Han P et al. *Cell* 2022*,* Kumar S et al. *Journal of medical virology* 2022, Lupala CS et al. *Biochemical and biophysical research communications* 2022 and Mannar D et al. *Science* 2022 *(See the reference list below). Table 2 i*n the revised version was shown for the detail residues and we also supplemented the reference together in the Table 2 below.**

**Reference:**

[1] Han P, Li L, Liu S, Wang Q, Zhang D, Xu Z, Han P, Li X, Peng Q, Su C, Huang B, Li D, Zhang R, Tian M, Fu L, Gao Y, Zhao X, Liu K, Qi J, Gao GF, Wang P. Receptor binding and complex structures of human ACE2 to spike RBD from omicron and delta SARS-CoV-2. Cell. 2022 Feb 17;185(4):630-640.e10.

[2] Celik, I., Yadav, R., Duzgun, Z., Albogami, S., El-Shehawi, A. M., Fatimawali, Idroes, R., Tallei, T. E., & Emran, T. B. (2021). Interactions of the Receptor Binding Domain of SARS-CoV-2 Variants with hACE2: Insights from Molecular Docking Analysis and Molecular Dynamic Simulation. *Biology*, *10*(9), 880.

[3] Kumar, S., Thambiraja, T. S., Karuppanan, K., & Subramaniam, G. (2022). Omicron and Delta variant of SARS-CoV-2: A comparative computational study of spike protein. Journal of medical virology, 94(4), 1641–1649.

[4] Lupala, C. S., Ye, Y., Chen, H., Su, X. D., & Liu, H. (2022). Mutations on RBD of SARS-CoV-2 Omicron variant result in stronger binding to human ACE2 receptor. *Biochemical and biophysical research communications*, *590*, 34–41.

[5] Mannar, D., Saville, J. W., Zhu, X., Srivastava, S. S., Berezuk, A. M., Tuttle, K. S., Marquez, A. C., Sekirov, I., & Subramaniam, S. (2022). SARS-CoV-2 Omicron variant: Antibody evasion and cryo-EM structure of spike protein-ACE2 complex. *Science (New York, N.Y.)*, *375*(6582), 760–764.

**Table 1 The active binding site of ACE2 and Delta spike protein complexes**

| RBD  (Delta) | ACE2 | references |
| --- | --- | --- |
| THR500 | TYR41 | [1][2] |
| GLY502 | LYS353 | [1][2] |
| GLN498 |  | [1][2] |
| GLY496 |  | [1][2] |
| GLY446 | GLN42 | [1][2] |
| TYR449 | ASP38 | [1][2] |
| GLN493 | LYS31  GLU35 | [1][2] |
|  |  | [2] |
| LYS417 | ASP30 | [1][2] |
| TYR489 | TYR83 | [1][2] |
| ASN487 | GLN24 | [1][2] |
| ALA475 | SER19 | [1][2] |

**Table 2 The active cores of ACE2 and Omicron spike protein complexes**

|  | RBD  (Omicron) | ACE2 | references |
| --- | --- | --- | --- |
| Site | ARG493 | Glu35 | [1][3][4] [5] |
|  | TYR453 | HIS34 | [1] |
|  | TYR501 |  | [3] |
|  | HIS505 | GLY354 | [1] |
|  | ASN477 | SER19 | [1] |
|  | SER496 | LYS353 | [1][5] |
|  | GLY502 |  | [1] [4] |
|  | THR500 | TYR41 | [1] |
|  | ARG498 | GLN42 | [1] [3] [5] |

7. Line129、132、137、146、147、148: resides/reside

**Response: Thank you very much for discovering these errors. We apologize for these grammatical problems and have corrected them based on your suggestions.**

8. Line 145-147,151-152: docking should be repeated and average scores should be given

**Response: Thank you for your suggestion. Following your advice, we have attached additional Table 3 to show all the scores getting from molecular docking assay. The average scores for Delta and Omicron with ACE2 were described in the Result part (Line146-147 and Line151-152).**

9. Line 149: better to give in kcal/mol units

**Response: Thank you for your correction. We have modified the unit.**

10. Line 147,152: There is no Table 3 in the text or supp.info

**Response: Thank you so much for your reminding. I'm very sorry that Table 3 was lost in the previous version. Here we have supplemented the missing information and numbered in Table 4 in the revised manuscript. Thank you so much for your reminding.**

11. Line 156-161: this sentence belongs to introduction.

**Response: Thank you for your comment. We have deleted the sentence in the Discussion Part, and the relative message in the sentence was then transferred to the Introduction Part.**

12. Line 192: This is not a virtual screening docking but just a molecular docking because only one molecular docking because only one molecule is tested against binding to certain targets

**Response: Thank you for your correction. We have revised the description “virtual molecular docking” to “molecular docking”.**

13. Line 195: Table 3

**Response: I'm very sorry that Table 3 was lost in the previous version. Here we have supplemented the missing information and numbered in Table 4 in the revised manuscript. Thank you so much for your reminding.**

14. Line 209-212: grammar should be corrected, computational results not mentioned.

**Response: Thank you very much for discovering these errors. We apologize for these grammatical problems. Furthermore, the conclusion was revised with supplementary computational results. The final summarized sentence is “**OA was identified as a novel active constituent which blocked both the binding of SARS-CoV-2 Delta (B.1.617.2) and Omicron (B.1.1.529) RBD to ACE2 *via* residues ARG403, GLY496 and LYS353 in Delta RBD-ACE2 complex and ARG403, HIS34 and LYS353 residues in Omicron RBD-ACE2 complex, suggesting the therapeutic candidate of OA for COVID-19 treatment.**”**

15. Line 227: No information on how many replicas were used. The error bar is wide in Fig2A Delta variant and needs to be explained and discussed.

**Response: Thank you so much for your remindings. N=3 was supplemented in Line 455 in the revised manuscript. And for the error bar in Fig 2A, according to your suggestion, we have checked the original data and found one of the values in OA in 100 mM group has been mis-inputted before analysis. We have re-corrected it and replaced the statistical analysis. The refreshed figure was presented in Fig 2A in the revised version.**

16. Line 261: “and” and “or” are used wrongly through out the text and the meanings are not clear.

**Response: Thank you for your correction. We have carefully checked the word “and” and “or” and some mis-using positions have been corrected. Please check.**

17. Line 277-278: How? To optimize the structure, the complex was initially processed by prepare protein” module in DS 2020, followed by supplementation of the missing sidechains, removement of the water molecules and protonation suitable at pH 7.4.

**Response: Thank you for the question. To optimize the structure, the buttons "clean protein" and "prepare protein" were clicked sequentially on the Discovery Studio software, the software will then automatically supplement missing sidechains of protein, remove the water molecules and protonate suitable in the chain at pH 7.4. It is a DS 2020 internal procedure which immediately response to the request required from a button instruct.**

18. Line 281: Needs to be more detailed, for example why the wt spike RBD was not used in docking studied to verify the experiments? Only the variants were used according to the results and methods do not address this issue
(1) Needs to be more detailed, for example why the wt spike RBD was not used in docking studied to verify the experiments?

**Response: Thank you for your comments. The details of docking were described started from Line 284 to 289.**

**All the related information mentioned here have been supplemented in the *Material and method* part in Red. Here the information are also shown below： Set the Input Ligands parameter to Molecule: All, and set the other parameters to the default values in“Prepare ligands” module.**

| **Parameter Name** | **Parameter Value** |
| --- | --- |
| **Input Ligands** | **Molecule: All** |
| **Change Ionization** | **True** |
| **Generate Tautomers** | **True** |
| **Generate Isomers** | **True** |
| **Fix Bad Valencies** | **True** |
| **Generate Coordinates** | **3D** |
| **Parallel Processing** | **False** |

(2) Only the variants were used according to the results and methods do not address this issue

**Response: Thank you for pointing the question. Yes, the variant Delta and Omicron were included in the assay instead of the wild type due to the low inhibitory activity of OA on WT RBD binding to ACE2 shown in Supplementary Figure 1 (see below as well). Therefore, we predicted the block effect of OA on wild type may not the main targets and all the assays in the manuscript were performed in variants not wild type.**

19. Line 290: Not clear, more details needed

**Response: Thank you for your suggestions. We have carefully supplemented the details of this section started from Line 291-300.**

20. Line 471-473: Mistakes in grammar

**Response: Thank you for your correction. We have carefully checked the word “and” and “or” and some mis-using positions have been corrected. Please check.**

21. Line 476: Molecular docking? There is no virtual screening

**Response: Thank you for your correction. Thank you for your correction. We have revised the description “virtual molecular docking” to “molecular docking”.**

22. Figure 7: side chains should be shown

**Response: Thank you for your request. The binding structures of RBD-ACE2 complex, particularly the active binding residues in the chains, have been fully shown in the Figure 7. Please check whether it fits.**
