# Supplementary material for: Oxalic acid blocked the binding of spike protein from SARS-CoV-2 Delta (B.1.617.2) and Omicron (B.1.1.529) variants to human angiotensin-converting enzymes 2
Source: PLoS One. 2023 May 18;18(5):e0285722. doi: 10.1371/journal.pone.0285722 (PMC10194883; doi:10.1371/journal.pone.0285722)
Supplement: Attachment — Submitted filename: Response to reviewers.docx [file pone.0285722.s004.docx]

1. In Tables 3 and 4, please add the minus (-) sign before the Interaction energy (kcal/mol) values. Positive interaction energy values are not acceptable, since they indicate an unfavorable interaction.

**Response: Thank you for your comments. Actually, in both Table 3 and 4, Interaction energy (kcal/mol) values were all below zero, and we also labelled value unit in the corresponding title (-Interaction energy). However, it may be not clear enough and may further confuse the readers. Following your question, we have revised the description of table title from ‘-Interaction energy’ to ‘Interaction energy’, and the values were thus presented with mimus in the following data. Please check.**

2. Please rephrase the sentence "When the variants have been keeping emerging during it spread, it is much smart and effective to target on the conservation receptor" between lines 182 - 183, as "As new variants continue to emerge during the spread of infection, it may be more rational and effective to target the ACE2 receptor."

**Response: Thank you for the suggestion and help. We have revised the description you mentioned here following your kindly provided. Please check.**
